# Preprocedural C-Reactive Protein Predicts Outcomes after Primary Percutaneous Coronary Intervention in Patients with ST-elevation Myocardial Infarction a systematic meta-analysis

**DOI:** 10.1038/srep41530

**Published:** 2017-01-27

**Authors:** Raluca-Ileana Mincu, Rolf Alexander Jánosi, Dragos Vinereanu, Tienush Rassaf, Matthias Totzeck

**Affiliations:** 1 University Hospital Essen, Medical Faculty, West German Heart and Vascular Center, Department of Cardiology and Vascular Diseases, Hufelandstr. 55, 45147 Essen, Germany; 2 University of Medicine and Pharmacy Carol Davila - University and Emergency Hospital, Cardiac Research Unit, Splaiul Independentei 169, 050098 Bucharest, Romania

## Abstract

Risk assessment in patients with acute coronary syndromes (ACS) is critical in order to provide adequate treatment. We performed a systematic meta-analysis to assess the predictive role of serum C-reactive protein (CRP) in patients with ST-segment elevation myocardial infarction (STEMI), treated with primary percutaneous coronary intervention (PPCI). We included 7 studies, out of 1,033 studies, with a total of 6,993 patients with STEMI undergoing PPCI, which were divided in the high or low CRP group, according to the validated cut-off values provided by the corresponding CRP assay. High CRP values were associated with increased in-hospital and follow-up all-cause mortality, in-hospital and follow-up major adverse cardiac events (MACE), and recurrent myocardial infarction (MI). The pre-procedural CRP predicted in-hospital target vessel revascularization (TVR), but was not associated with acute/subacute and follow-up in-stent restenosis (ISR), and follow-up TVR. Thus, pre-procedural serum CRP could be a valuable predictor of global cardiovascular risk, rather than a predictor of stent-related complications in patients with STEMI undergoing PPCI. This biomarker might have the potential to improve the management of these high-risk patients.

Coronary artery disease (CAD) is the most common cause of death worldwide, with an overall mortality of over 7 million people per year^1^. Inflammation plays an important, but yet incompletely defined role in CAD and in ACS, particularly by contributing to plaque rupture and erosion, which precedes the formation of the overlying thrombosis^2^,^3^. The degree of the thrombus blockage determines the type of the ACS: unstable angina (UA), with partial or intermittent coronary artery occlusion and no myocardial injury; non-ST-elevation myocardial infarction (NSTEMI), with partial or intermittent coronary artery occlusion with myocardial damage, and elevated circulating troponin levels; and STEMI, with complete coronary artery occlusion with myocardial damage, and changes in electrocardiogram^4^,^5^. The mortality of STEMI patients is about 12% at 6 months, with higher mortality rates in high-risk individuals. Despite all attempts to improve therapeutic approaches, patients with STEMI continue to have a limited prognosis^6^,^7^ and it is important to identify new markers that predict the outcomes in this patient cohort.

CRP is an acute phase reactant produced by hepatocytes in reaction to pro-inflammatory cytokines. Elevated CRP levels have been associated with a decrease in endothelial nitric oxide (NO) production^8^ and an upregulation in endothelin-1 generation, a potent vasoconstrictor produced by the endothelial cells. This causes endothelial dysfunction, which is the hallmark for arteriosclerosis. Furthermore, the expression of chemokines and adhesion proteins^9^ is promoted. CRP is considered a risk factor for cardiovascular disease, with the relative risk bordering on those of classical risk factors, such as LDL-cholesterol, arterial hypertension or smoking^10^,^11^,^12^,^13^,^14^. Several large population studies have demonstrated that high levels of CRP could be an outcome predictor in patients undergoing elective percutaneous coronary intervention (PCI) for stable coronary artery disease^15^,^16^, non-ST-elevation acute coronary syndromes^17^,^18^ or mixed populations^19^,^20^,^21^,^22^,^23^. However, only few evidences are available regarding the role of CRP as a predictor of outcomes in STEMI patients treated by primary percutaneous coronary intervention (PPCI).

According to the current guidelines, PPCI is the gold standard for the treatment of STEMI patients^1^,^24^. PPCI is defined as the PCI in the setting of STEMI, without previous fibrinolysis, and it is indicated in all patients with STEMI in the first 12 hours from symptom onset^1^. Compared to fibrinolysis, PPCI results in higher rates of infarct-related artery patency, higher rates of myocardial blush and lower rates of complications, such as recurrent ischemia, reinfarction, emergency repeat revascularization procedures, intracranial hemorrhage or death^25^. After revascularization with PPCI, STEMI patients require a special management. Although the last decades provided tremendous advance in the management of STEMI, the mortality is still high and the management is very expensive. Pre-procedural CRP monitoring could be of use in identifying high-risk patients and guiding the management of the STEMI patients, in order to improve their outcome. We performed a systematic meta-analysis in order to assess the predictive role of serum CRP on in-hospital and follow-up outcomes, in patients with STEMI treated with PPCI.

## Results

### Study selection

**Figure 1:**
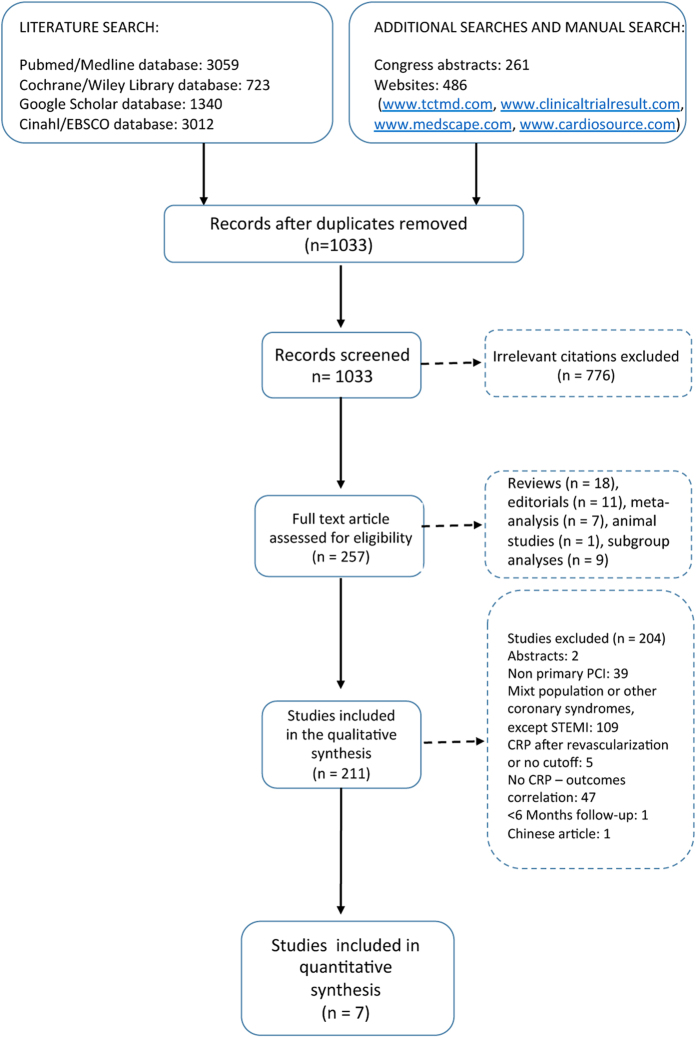
PRISMA selection flowchart^47^.

1,033 studies were screened after removing the duplicates from the total amount of papers, 776 irrelevant citations were excluded, 257 full text articles were assessed for eligibility. 46 studies were excluded because they were either reviews, editorials, unrelated meta-analysis, animal studies or subgroup analyses. 204 studies were excluded because they did not meet the inclusion criteria: 2 studies were presented as abstracts, 39 studies did not evaluate PPCI, 109 studies contained mixed populations or other coronary syndromes except for STEMI, 5 studies determined CRP after revascularization or provided no CRP cut-off, 47 studies presented no CRP-outcomes correlation, one study did have a follow-up under 6 months and one study was in Chinese. Consequently, 7 studies were included in our meta-analysis, 6 retrospective studies^26^,^27^,^28^,^29^,^30^,^31^ and 1 prospective cohort study^32^. The study selection process is shown in Fig. 1. Overall, there were 6,993 patients involved in our analysis, 5,225 included in the low CRP group and 1,768 in the high CRP group. The follow-up period varied between 6 months and 36 months. The characteristics of the selected studies are shown in Table 1. The quality of the included studies was high, with 6 to 8 stars out of a maximum of 9, according to the Newcastle-Ottawa Scale (Table 2). The CRP was assessed by highly sensitive assays methods in all studies, except for Tomoda *et al*.^28^. The cut-off value was below 1 mg/dl and defined to be 0.2 mg/dl in one study^27^, 0.3 mg/dl in three studies^26^,^28^,^31^, 0.5 mg/dl in two studies^30^,^32^, and 0.7 mg/dl in one study^29^.

### CRP and in-hospital and follow-up all-cause mortality

**Figure 2:**
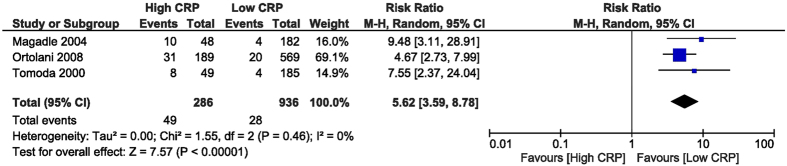
Overall and each study estimate of the RR of in-hospital all-cause mortality associated with high vs. low levels of CRP. Square boxes denote the RR, horizontal lines represent 95% confidence intervals. Weights are from random effects analysis. RR = risk ratio, CRP = C-reactive protein.

**Figure 3:**
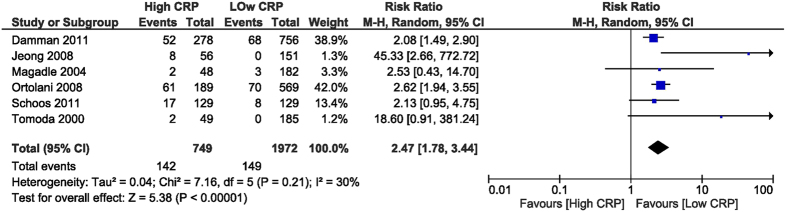
Overall and each study estimate of the RR of follow-up all-cause mortality associated with high vs. low levels of CRP. Square boxes denote the RR, horizontal lines represent 95% confidence intervals. Weights are from random effects analysis. RR = risk ratio, CRP = C-reactive protein.

High CRP was associated with increased in-hospital all-cause mortality, with a RR of 5.62 (95% CI [3.59, 8.78], p < 0.001) assessed from 3 studies^26^,^28^,^32^ reporting this outcome, including 1,222 patients (Fig. 2). The specific causes of death were not described and this is why we called it all-cause mortality. The follow-up all-cause mortality was increased in the high CRP group, with a RR of 2.47 (95% CI [1.78, 3.44], p < 0.001), as obtained from 6 studies^26^,^27^,^28^,^29^,^30^,^32^ which reported this outcome, including 2,721 patients (Fig. 3).

### CRP and major adverse cardiac events (MACE)

**Figure 4:**
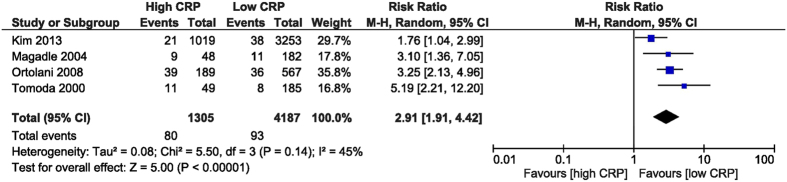
Overall and each study estimate of the RR of in-hospital MACE associated with high vs. low levels of CRP. Square boxes denote the RR, horizontal lines represent 95% confidence intervals. Weights are from random effects analysis. RR = risk ratio, CRP = C-reactive protein, MACE = major adverse cardiac events.

**Figure 5:**
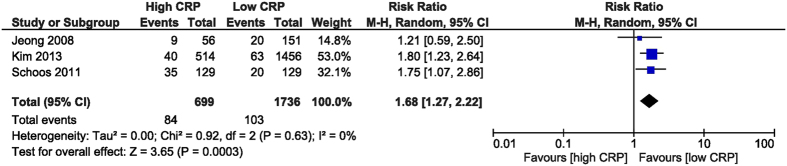
Overall and each study estimate of the RR of follow-up MACE associated with high vs. low levels of CRP. Square boxes denote the RR, horizontal lines represent 95% confidence intervals. Weights are from random effects analysis. RR = risk ratio, CRP = C-reactive protein, MACE = major adverse cardiac events.

The in-hospital MACE were increased in the high CRP group, with a RR of 2.91 (95% CI [1.91, 4.42], p < 0.001). The RR was obtained from 4 studies^26^,^28^,^31^,^32^ which reported this outcome, including 5,492 patients. MACE was defined as a composite of death, target vessel revascularization, recurrent myocardial infarction (MI), and stent reocclusion (Fig. 4). The follow-up MACE RR was 1.68 (95% CI [1.27, 2.22], p < 0.001) after analysing 2,435 patients from 3 studies^27^,^30^,^31^ who reported this outcome (Fig. 5).

### CRP and recurrent MI

**Figure 6:**
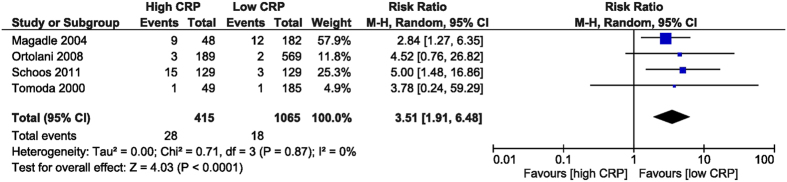
Overall and each study estimate of the RR of recurrent MI associated with high vs. low levels of CRP. Square boxes denote the RR, horizontal lines represent 95% confidence intervals. Weights are from random effects analysis. CRP = C-reactive protein, MI = myocardial infarction.

The recurrent MI risk was increased in the high CRP group, RR was 3.51 (95% CI [1.91, 6.48], p < 0.001) obtained from 4 studies^26^,^27^,^28^,^32^ which reported this outcome, including 1,480 patients (Fig. 6).

### CRP and acute/subacute in-stent restenosis (ISR)

**Figure 7:**
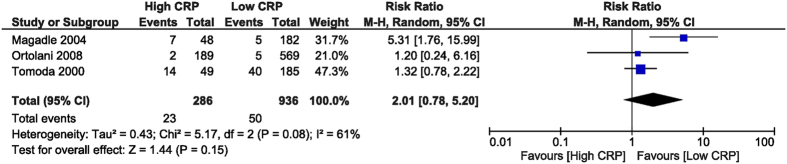
Overall and each study estimate of the RR of acute/subacute ISR associated with high vs. low levels of CRP. Square boxes denote the RR, horizontal lines represent 95% confidence intervals. Weights are from random effects analysis. RR = risk ratio, CRP = C-reactive protein, ISR = in-stent restenosis.

**Figure 8:**
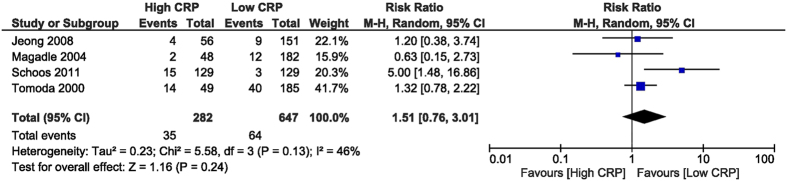
Overall and each study estimate of the RR of follow-up ISR associated with high vs. low levels of CRP. Square boxes denote the RR, horizontal lines represent 95% confidence intervals. Weights are from random effects analysis. RR = risk ratio, CRP = C-reactive protein, ISR = in-stent restenosis.

Acute/subacute in-stent restenosis was not different between groups, with RR of 2.01 (95% CI [0.78, 5.2], p = 0.15) derived from analysing 3 studies^26^,^28^,^32^ that reported this outcome, with 1,222 patients (Fig. 7). The follow-up restenosis was not different between the high and low CRP groups, with RR of 1.51 (95% CI [0.76, 3.01], p = 0.24) extracted from 4 studies^27^,^28^,^30^,^32^ with 929 patients (Fig. 8).

### CRP and in-hospital target vessel revascularization (TVR)

**Figure 9:**
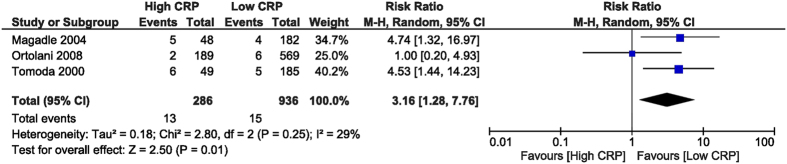
Overall and each study estimate of the RR of in-hospital TVR associated with high vs. low levels of CRP. Square boxes denote the RR, horizontal lines represent 95% confidence intervals. Weights are from random effects analysis. RR = risk ration, CRP = C-reactive protein, TVR = target vessel revascularization.

**Figure 10:**
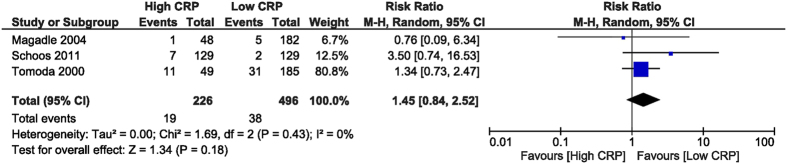
Overall and each study estimate of the RR of follow-up TVR associated with high vs. low levels of CRP. Square boxes denote the RR, horizontal lines represent 95% confidence intervals. Weights are from random effects analysis. RR = risk ratio, CRP = C-reactive protein, TVR = target vessel revascularization.

In-hospital TVR was increased in the high CRP group, with a RR of 3.16 (95% CI [1.28, 7.76], p = 0.01). To obtain this end-point we analysed data from 3 studies^26^,^28^,^32^ with 1,222 patients. The TVR was defined as coronary arterial by-pass surgery or PCI of the culprit vessel (Fig. 9). The follow-up TVR was similar between the two groups, with an RR of 1.45 (95% CI [0.84, 2.52], p = 0.18) derived from 3 studies^27^,^28^,^32^ which reported this outcome, with 722 patients (Fig. 10).

### Heterogeneity between studies, inconsistency and publication bias

**Figure 11:**
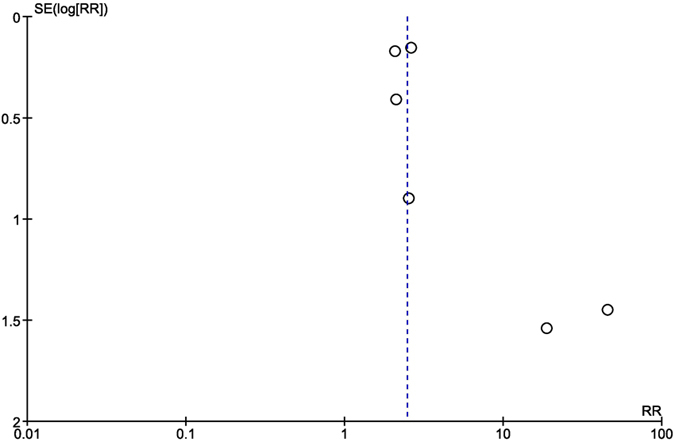
Funnel plot for publication bias in overall effect publication, measured as SE of log RR, against the treatment effect log RR. SE(log[RR]) = standard error of log relative risk.

There was no significant heterogeneity between studies and the inconsistency was significant in the acute/subacute ISR analysis, where I^2^ = 61% (Fig. 7). The publication bias was not significant, as assessed by the Egger’s test (Fig. 11).

### The sensitivity and subgroup analysis

A sensitivity analysis was performed to address the relative importance of each study, by excluding each study in turn from the analysis. The predictive value of the CRP level maintains for all outcomes. The predictive value of CRP persists when performing the subgroup analysis and comparing the studies with the same CRP cut-off values.

## Discussion

This meta-analysis assessed the predictive power of pre-procedural CRP level for short- and long-term outcomes in patients with STEMI treated with PPCI. The study pooled 7 studies, including 6,993 patients.

The main findings of this meta-analysis are:

1. Patients with high pre-procedural CRP level have a statistically significant increase in in-hospital and follow-up all-cause mortality, in-hospital and follow-up MACE, and recurrent MI.
2. Pre-procedural CRP predicts in-hospital TVR, which is important in the emergency setting, but has no predictive value for the acute/subacute and follow-up ISR, and follow-up TVR.

Many studies assessed the role of CRP in predicting cardiovascular outcomes, but there were no consistent data on the assessment of the CRP predictive value in STEMI patients undergoing PPCI.

The current European Society of Cardiology guidelines do not advise a routinely measurement of CRP, neither in the management of ACS patients^1^,^33^, nor in prevention. They indicate that CRP level could improve the risk stratification and could be useful in the management of the statin treatment^34^. The American Heart Association guideline indicates the measurement of serum CRP to assist risk-based treatment decisions^35^. Our study findings suggest that CRP might be of tremendous importance in the development of an individual-risk approach in STEMI patients undergoing PPCI.

Our findings are in line with one study^20^ that assessed the predictive value of CRP in patients undergoing elective PCI and showed that high pre-procedural CRP levels were associated with a higher risk of mortality or MI, but are not related to target vessel revascularization or stent thrombosis. Another study^21^ on more than 8,800 patients defined CRP as a predictor of all-cause mortality in patients undergoing elective PCI, independent of the LDL cholesterol value. In patients with coronary artery disease undergoing all types of PCI, baseline CRP level predicts one-year mortality and MACE^15^,^22^, result which is concordant with our findings. A comprehensive meta-analysis^23^, including over 34,000 patients that underwent PCI for different conditions, showed that high CRP levels were associated with increased MACE, all-cause mortality, myocardial infarction, coronary revascularization, and clinical restenosis, and concluded that every 1 mg/L in the CRP value was associated with 12% increase in the risk of MACE. There are also studies^36^ that did not find any association between the risk of stent restenosis after drug-eluting stents (DES) implantation and CRP, which is similar with our findings. On the other side, a meta-analysis^37^ that included over 2,700 patients undergoing all types of PCI with bare-metal stents (BMS), but not defining subgroups of PPCI, showed that higher baseline CRP levels are associated with higher risk of angiographic restenosis. In the same direction, one study^38^ showed that patients with CRP < 0.3 mg/dl after follow-up angiography after DES implantation, had a lower risk of MACE and restenosis rate. A meta-analysis^39^ on 1,062 patients, showed that elevate pre-procedural CRP is associated with greater in-stent restenosis after stenting, with greater impact in unstable-angina patients. So the importance of pre-procedural CRP in predicting stent-related outcomes remains uncertain.

CRP has gained interest as a marker of risk stratification in acute coronary syndromes^40^, but the most important question would be if this information may influence clinical practice. We have chosen a high-risk group of patients in our meta-analysis, because it is of paramount importance to improve the risk assessment in this group and to tailor the treatment options on the patient’s individual risk. The most important clinically applicable outputs arise from the statin trials, and are based on the pleiotropic anti-inflammatory effect of statins, that reduces the CRP level and consequently improves the prognosis^41^,^42^,^43^,^44^. Current evidence shows a fundamental role of inflammation in all stages of the atherosclerotic process^45^,^46^, but the measures to reduce inflammation have not been yet translated into clinical practice. Thus, our meta-analysis contributes to the potential development of new management protocols of patients with STEMI that undergo PPCI, by selecting, according to the value of CRP, the high risk patients.

### Study limitations

Our meta-analysis has some limitation that should be addressed. Firstly, the publication bias may impact the final result, the studies included in the analysis were longitudinal studies, most of them retrospective, and not randomized trial, because there were no randomized controls studies performed regarding our studied population. However, the longitudinal studies reflect the clinic reality and they are useful in decision making. Secondly, the different cut-off values of CRP and the different methods of assessment between studies could be a limitation, as well as the different follow-up times. Thirdly, there was no uniform definition of MACE across the studies.

## Conclusion

Pre-procedural serum CRP could be a valuable predictor of the global cardiovascular risk, rather than a predictor of stent-related complications in patients with STEMI undergoing PPCI. This biomarker could help to improve the management of these high-risk patients. The clinical application of determining CRP value before PPCI appears promising, but warrants confirmation by large, well-designed prospective and randomized trials.

## Methods

The methods used to perform this work were in compliance with the PRISMA (Preferred Reporting of Items for Systematic Meta-Analysis) statement for studies that evaluate health care interventions^47^.

### Information sources and search strategies

A systematic search of studies published until August 2016 was performed through MEDLINE, Cochrane, EMBASE, and Google Scholar databases, through the major cardiology websites (www.tctmd.com, www.clinicaltrialresult.com, www.medscape.com, www.cardiosource.com), and through the abstracts or presentations of annual meetings of the major cardiovascular societies (European Society of Cardiology and its branches, American Heart Association, American College of Cardiology, Society of Cardiovascular Angiography and Intervention, Transcatheter Cardiovascular Therapeutics, and China Interventional Therapeutics).

We made our search specific and sensitive using the MeSH (Medical Subject Headings) terms (Table 3) and free text. We considered studies in any language. Supplementary Table 1 describes the search result trough Medline performed on the 8^th^ of August 2016.

### Inclusion criteria

Studies that fulfilled all the criteria below were included:

1. Randomized studies, prospective or retrospective observational design studies.
2. Patients with STEMI that undergone PPCI.
3. Blood samples for CRP were collected before revascularization and cut-off values for CRP were provided.
4. Minimum 6 Months follow-up.

### Exclusion criteria

1. Subgroup studies, review studies, animal studies, laboratory studies, abstracts.
2. Patients that undergone PCI for other pathology (not PPCI) or mixed population without reported outcomes in the PPCI subset.
3. Blood samples collected after revascularization.
4. No relation between CRP value and clinical outcomes.

### Data extraction and quality assessment

Two of the authors (RIM and MT) independently performed data extraction, using a standard data extraction form that contained publication details (name of the first author, year of publication), study design, characteristics of the studied population (sample size, gender distribution), methods of CRP measurement, CRP cut-off, duration of follow-up, and outcomes.

Two of the authors (RIM and MT) assessed independently the trial eligibility, the trail quality, and extracted the data. The trial quality was assessed using the Newcastle-Ottawa Scale^48^, because the Cochrane Handbook^49^ risk of bias refers especially to randomised trials. According to this scale, each study is judged on eight items, categorized into three groups: the selection of the study groups, the comparability of the groups, and the ascertainment of either the exposure or outcome of interest for case-control or cohort studies respectively. A maximum of 4 stars for selection, 2 stars for comparability, and 3 stars for outcomes could be awarded. Stars are awarded such that the highest quality studies are awarded up to 9 stars. The guidelines for reporting the meta-analysis of observational studies^50^ recognizes that the use of quality scoring in meta-analysis of observational studies is controversial and recommends the reporting of quality scoring, if it has been done, and subgroup or sensitivity analysis, rather than using the quality scores.

### Study endpoints

The endpoints were: in-hospital and follow-up all-cause mortality, in-hospital and follow-up MACE, recurrent MI, acute or subacute ISR and follow-up ISR, in-hospital and follow-up TVR. MACE were defined as a composite of death, target vessel revascularization, recurrent MI, and stent reocclusion. TVR was defined as coronary arterial by-pass surgery or PCI of the culprit vessel. One study^26^ reported the outcomes in quartiles of CRP and we considered the first three quartiles as low CRP group, because the CRP value was <0.3 mg/dl and the forth quartile as the high CRP group. In one study^27^ we considered the total event rate according to the CRP cut-off, irrespective of the stent type. In one study^31^ we considered the total event rate according to the cut-off value of CRP, without taking into consideration the symptoms-to-balloon time.

### Statistical analysis

The meta-analysis was conducted for eligible studies as per risk estimates by two categories: low CRP values and high CRP values. Data are expressed as RR and 95% confidence interval (95% CI) for dichotomous outcomes^51^. The cut-off value for the high CRP was considered according to the validated cut-off values provided by the corresponding CRP assay. We included in the high CRP group all patients with CRP values above the cut-off provided by the manufacturer of the CRP assay (see Table 1), according to the calibration tests, while the rest of patients were included in the low CRP group. A random-effect, rather than a fixed-effect was adopted, because this is likely the most appropriate and conservative, accounting for differences among trials. Heterogeneity between studies was assessed by Q statistic and inconsistency was quantified with the I^2^ statistic. Because this test has a poor power in the event of few studies, we considered both the presence of significant heterogeneity at the 10% level of significance and value of I^2^ ≥56% as an indicator of significant heterogeneity^52^. The presence of publication bias was assessed by Egger’s test^53^. All analyses were conducted using Review Manager version 5.3 (Revman, The Cochrane Collaboration, Oxford, United Kingdom).

## Additional Information

**How to cite this article**: Mincu, R.-I. *et al*. Preprocedural C-Reactive Protein Predicts Outcomes after Primary Percutaneous Coronary Intervention in Patients with ST-elevation Myocardial Infarction a systematic meta-analysis. *Sci. Rep.* **7**, 41530; doi: 10.1038/srep41530 (2017).

**Publisher's note:** Springer Nature remains neutral with regard to jurisdictional claims in published maps and institutional affiliations.

## Supplementary Material

Supplementary Table 1

## Figures and Tables

**Table 1:** Characteristics of the studies included in the meta-analysis.

First author & year	Design	Number of patients included N (% males)	CRP assay	CRP cut-off (mg/dl)	Follow-up duration (months)
Ortolani^26^	Retrospective	758 (70)	Highly sensitive nephelometry	0.31	36
Schoos^27^	Retrospective	258 (76)	Highly sensitive immunoturbidimetric analysis	0.2	36
Tomoda^28^	Retrospective	234 (77)	Latex photometric immunoassay	0.3	6
Damman^29^	Retrospective	1034 (73)	Highly sensitive immunoturbidimetric assay	0.7	30
Jeong^30^	Retrospective	207 (81)	Highly sensitive immunoturbidimetric assay	0.5	12
Kim^31^	Retrospective	4272 (74)	Highly sensitive immunoturbidimetric assay	0.3	36
Magadle^32^	Cohort	230 (77)	Highly sensitive latex enhanced nephelometry	0.5	12

**Table 2:** Quality assessment of the included studies using the Newcastle-Ottawa Scale^48^.

Study	Ortolani 2008	Schoos 2011	Tomoda 2000	Magadle 2004	Jeong 2008	Damman 2011	Kim 2013
Is the selected cohort representative?	Yes	Yes	Yes	Yes	Yes	Yes	Yes
Is the selection of controls appropriate?	Yes	Yes	Yes	Yes	Yes	Yes	Yes
Is the ascertainment of exposure appropriate?	Yes	Yes	Yes	Yes	Yes	Yes	Yes
Is the demonstration that outcome of interest was not present at the start of the study true?	Yes	Yes	Yes	Yes	Yes	Yes	Yes
Are the selected and control groups comparable concerning age/other controlled factors?	No/No	Yes/No	Yes/Yes	Yes/Yes	No/No	Yes/No	No/No
Is the independent or blind assessment stated in the paper?	Yes	Yes	No	No	No	No	No
Was follow-up long enough?	Yes	Yes	No	Yes	Yes	Yes	Yes
Was follow-up adequate?	Yes	Yes	Yes	Yes	Yes	Yes	Yes
Total number of stars	7	8	7	8	6	7	6

Yes = one star, no = no star.

**Table 3:** MeSH terms used for search.

Term	MeSH terms
ST segment elevation myocardial infarction	myocardial infarction, coronary disease, acute coronary syndrome
Primary PCI	angioplasty, balloon, coronary, percutaneous coronary intervention, drug-eluting stents, self-expandable metallic stents
C reactive protein	C-reactive protein, acute-phase reaction

MeSH = Medical Subject Headings, PCI = percutaneous coronary intervention.
