# Supplementary material for: Preprocedural C-Reactive Protein Predicts Outcomes after Primary Percutaneous Coronary Intervention in Patients with ST-elevation Myocardial Infarction a systematic meta-analysis
Source: Sci Rep. 2017 Jan 27;7:41530. doi: 10.1038/srep41530 (PMC5270244; doi:10.1038/srep41530)
Supplement: Supplementary Table 1 [file srep41530-s1.pdf]

PREPROCEDURAL C-REACTIVE PROTEIN PREDICTS OUTCOMES AFTER PRIMARY PERCUTANEOUS CORONARY INTERVENTION IN PATIENTS WITH ST-ELEVATION MYOCARDIAL INFARCTION

a systematic meta-analysis

Raluca-Ileana Mincu[1,2], Rolf Alexander Jánosi[1], Dragos Vinereanu[2], Tienush Rassaf[1], and Matthias Totzeck[1*]

[1]University Hospital Essen, Medical Faculty, West German Heart and Vascular Center, Department of Cardiology and Vascular Diseases, Hufelandstr. 55, 45147 Essen, Germany

[2]University of Medicine and Pharmacy Carol Davila - University and Emergency Hospital, Cardiac Research Unit, Splaiul Independentei 169, 050098 Bucharest, Romania

*Corresponding author
Dr. Matthias Totzeck
University Hospital Essen
West German Heart and Vascular Center
Clinic for Cardiology and Vascular Diseases
Hufelandstr. 55, 45147 Essen, Germany
Tel.: +49 201 723 84805
Fax: +49 201 723 5401
Email: Matthias.Totzeck@uk-essen.de

Supplementary table 1.

The results of the search through Medline on the 8th of August 2016

Nr of search	Query	Medline
1	myocardial infarction AND percutaneous coronary intervention AND C reactive protein	451
2	acute myocardial infarction AND percutaneous coronary intervention AND C reactive protein	283
3	acute myocardial infarction AND percutaneous coronary intervention AND CRP	142
4	acute myocardial infarction AND percutaneous coronary intervention AND acute-phase reaction	11
5	myocardial infarction AND angioplasty AND C reactive protein	306
6	myocardial infarction AND drug-eluting-stent AND C reactive protein	41
7	myocardial infarction AND stent AND C reactive protein	138
8	STEMI AND percutaneous coronary intervention AND C reactive protein	115
9	acute coronary syndrome AND percutaneous coronary intervention AND C reactive protein	167
10	acute coronary syndrome AND stent AND C reactive protein	47
11	coronary disease AND stent AND C reactive protein	294
12	coronary disease AND percutaneous coronary intervention AND C reactive protein	501
13	STEMI AND stent AND C reactive protein	17
14	STEMI AND angioplasty AND C reactive protein	50
15	acute coronary syndrome AND angioplasty AND C reactive protein	108
16	coronary disease AND angioplasty AND C reactive protein	388
